# Thirdhand vaping exposures are associated with pulmonary and systemic inflammation in a mouse model

**DOI:** 10.20517/jeea.2023.27

**Published:** 2023-10-29

**Authors:** Sarah Commodore, Shikha Sharma, Carolyn Damilola Ekpruke, Robert Pepin, Angela M. Hansen, Dustin Rousselle, Maksat Babayev, Jonas M. Ndeke, Rachel Alford, Erik Parker, Stephanie Dickinson, Sunita Sharma, Patricia Silveyra

**Affiliations:** 1 Department of Environmental and Occupational Health, School of Public Health Bloomington, Indiana University, Bloomington, IN 47408, USA.; 2 Department of Chemistry, Indiana University, Bloomington, IN 47405, USA.; 3 Department of Epidemiology and Biostatistics, School of Public Health Bloomington, Indiana University, Bloomington, IN 47405, USA.; 4 Biostatistics Consulting Center, Department of Epidemiology and Biostatistics, School of Public Health Bloomington, Indiana University, Bloomington, IN 47405, USA.; 5 Division of Pulmonary Sciences and Critical Care Medicine, University of Colorado Anschutz Medical Campus, Aurora, CO 80045, USA.; 6 Department of Medicine, Indiana University School of Medicine, Indianapolis, IN 46202, USA.

**Keywords:** E-cigarettes, blood cytokines, lung cytokines, mouse model, particulate matter, organic chemicals, thirdhand smoke, vaping

## Abstract

Thirdhand smoke (THS) is the accumulation of secondhand smoke on surfaces that ages with time. THS exposure is a potential health threat to children, partners of smokers, and workers in environments with current or past smoking, and needs further investigation. In this study, we hypothesized that thirdhand Electronic Nicotine Delivery Systems (ENDS) exposures elicit lung and systemic inflammation due to resuspended particulate matter (PM) and inorganic compounds that remain after active vaping has ceased. To test our hypothesis, we exposed C57BL/6J mice to cotton towels contaminated with ENDS aerosols from unflavored vape fluid (6 mg nicotine in 50/50 propylene glycol/vegetable glycerin) for 1h/day, five days/week, for three weeks. We assessed protein levels in serum and bronchoalveolar lavage fluid (BALF) using a multiplex protein assay. The mean ± sd for PM_10_ and PM_2.5_ measurements in exposed mouse cages were 8.3 ± 14.0 and 4.6 ± 7.5 μg/m^3^, compared to 6.1 ± 11.2 and 3.7 ± 6.6 μg/m^3^ in control cages respectively. Two compounds, 4-methyl-1, 2-dioxolane and 4-methyl-cyclohexanol, were detected in vape fluid and on ENDS-contaminated towels, but not on control towels. Mice exposed to ENDS-contaminated towels had lower levels of serum Il-7 (*P* = 0.022, *n* = 7), and higher levels of Il-13 in the BALF (*P* = 0.006, *n* = 7) than those exposed to control towels (*n* = 6). After adjusting for sex and age, Il-7 and Il-13 levels were still associated with thirdhand vaping exposure (*P* = 0.010 and *P* = 0.017, respectively). This study provides further evidence that thirdhand ENDS aerosols can contaminate surfaces, and subsequently influence lung and systemic health upon exposure.

## INTRODUCTION

The rising use of ENDS is considered a significant and emerging public health problem^[1]^. ENDS represent a diverse class of products, including electronic cigarettes, vapes, vaporizers, vape pens, hookah pens, and pods^[2]^. Recently, the adoption of disposable/single-use ENDS has increased^[3]^, most probably due to the United States Food and Drug Administration’s (FDA) prioritized enforcement against flavored cartridge-based ENDS products^[4]^. For instance, the percentage of disposable e-cigarette sales for non-menthol cooling flavors increased from 5.2% in 2017 to 99.2% in 2021^[5]^. In 2021, 4.5% of adults in the US were current e-cig users, and among these, 60% of users aged 18 to 24 years were previously nonsmokers^[6]^. Regardless of ENDS products including rechargeable and disposable devices, new complex compounds with unknown health consequences could be formed during and after use. The toxicity of these potentially harmful compounds and their effects on lung and systemic health have not yet been investigated.

Unique subgroups such as nonsmokers and children who encounter surfaces contaminated with ENDS vapors can be exposed to a hidden environmental risk factor, namely THS^[7]^. THS from traditional cigarette smoking is a recognized public health hazard^[8]^, and is associated with adverse health effects in multiple organ systems^[9,10]^. Similarly, ENDS aerosols can be a potential source of thirdhand exposure^[11]^. Epidemiological, *in vivo*, and *in vitro* studies suggest that ENDS aerosol exposures are not harmless and can cause molecular, cellular, and physiological alterations, such as impaired lung function and inflammation^[12]^. Such health effects are analogous to traditional cigarette smoke exposures^[13]^. Even a 30-min exposure to passive ENDS aerosols is associated with alterations in respiratory mechanics and exhaled biomarkers^[14]^. Yet, the health effects of long-term thirdhand ENDS exposures remain unknown.

As of October 2023, there have been only two experimental studies using a BALB/c mouse model to investigate thirdhand ENDS exposure effects^[15,16]^. In the current study, we sought to complement the previous work by using a mouse model on the C57BL/6 background to expand the current understanding of thirdhand vaping exposures and evaluate some potential implications for humans. Thus, the motivating hypothesis for this work is that thirdhand ENDS exposures elicit lung and systemic inflammation, as a result of differential PM concentrations for C57BL/6J mice compared to controls. To address this hypothesis, we sought to (1) assess PM concentrations of an ENDS-contaminated material after active vaping has ceased; (2) identify organic chemicals found on materials contaminated with ENDS aerosols compared to control materials; (3) examine whether thirdhand ENDS exposure differentially affected indicators of health such as proteins in serum and bronchoalveolar lavage fluid of the exposed mice compared to control mice.

## MATERIALS AND METHODS

### ENDS aerosol generation

**Figure 1.**
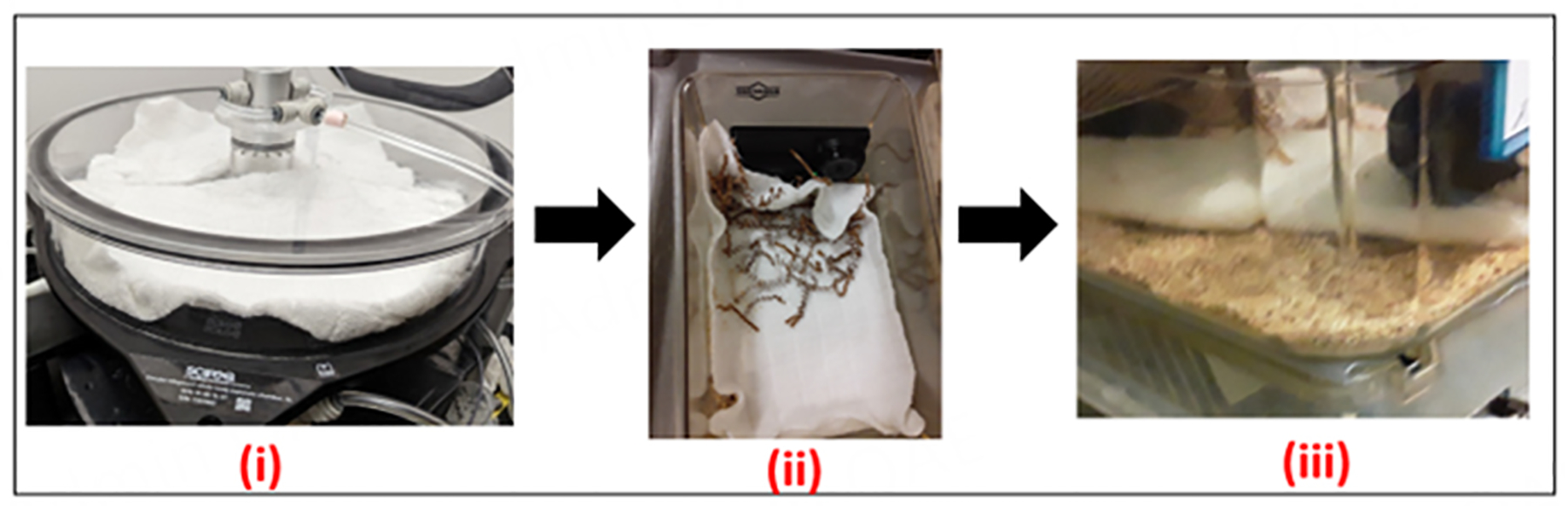
The sample chamber of the inhalation exposure system with aerosol generated onto towels (i); To assess differences in PM concentrations, UPAS V2+ air samplers were placed in a subset of exposed and control mouse cages in (ii); Finally, ENDS-contaminated and control towels were placed in clean mouse cages, and mice were placed on towels 1hr/day, 5 days a week over a three-week period (iii). ENDS: electronic nicotine delivery systems; PM: particulate matter.

To generate ENDS-contaminated towels, we exposed a new towel (12 cm × 10 cm, 100% white cotton) to aerosols generated by 1 puff/minute at a volume of 70 mL and a duration of 3.3s bursts of ENDS inside a SCIREQ^®^ inExpose^™^ system (230 °C and resistance of 1.5 ohm) for one hour. The inExpose^™^ system is a computerized inhalation machine that allows control of exposure doses of nicotine and the exposure duration to cigarettes/e-cigarettes^[17]^ [Figure 1]. The vape fluid (BN E25214) was purchased from Vapor Vapes Inc (Sand City, CA) and was unflavored. The listed ingredients on the bottle were propylene glycol (PG), USP Grade Vegetable Glycerin (VG), Liquid Nicotine, and FDA-approved flavors. The vape fluid used for this experiment had 6mg of nicotine with a 50/50 mix of PV/VG.

### Animals

Male and female wild-type mice (8 to 10 weeks of age) from the C57BL/6 background were purchased from JAX laboratories (Bar Harbor, ME) and housed, bred, and maintained in a 12/12h light/dark cycle with food and water available ad libitum. The Indiana University Bloomington Institutional Animal Care and Use Committee (IACUC) approved all procedures (protocol #22–026). The institution is accredited by the Association for Assessment and Accreditation of Laboratory Animal Care (AAALAC).

Mice of two different ages were used in this study. The first were 4-weeks old and consisted of five males from the same litter. Three of these juveniles were assigned to the thirdhand ENDS exposure and two to the control. The second category of mice was 8 weeks old (four males and four females, also from the same litter). For this second group, two males and two females were assigned to the thirdhand ENDS exposure, and two males and two females were assigned to control towels. Thus, a total of seven mice (five males and two females) were exposed to ENDS-contaminated towels and a total of six mice (four males and two females) were exposed to control towels.

### Thirdhand exposure

To expose mice to thirdhand ENDS aerosols, mice were removed from their home cages and moved to an experimental clean cage. The bottom of the mouse cage was lined with 100% cotton towels. These towels were purchased from a local department store (brand = room essentials, with the following tag: RN 17730, VN 1120066, F16827389, Q4/19L9897) and cut into 12 cm × 10 cm pieces. Approximately half of these cut towels were exposed to ENDS aerosols. Each exposure lasted for one hour and was repeated daily (5 times/week) for 3 weeks. The schedule included daily thirdhand ENDS aerosol exposures for five consecutive days, followed by two days off. A new towel was used for each set of 5-day exposure period. So, for each exposure group, there were three total towel samples by the end of the three-week exposure period. Control mice were exposed to 100% cotton towels with no previous ENDS aerosol contamination.

### Environmental conditions during exposure

During the one-hour exposure period, animals were housed in the same way as their home [static] cages (e.g., if there are two or three animals, all two or three will be put in the experimental cage for the exposure period). Control animals had their cages lined with the same type of towel, but this towel was not previously exposed to ENDS aerosols. Animals had access to food and water *ad libitum* during this time as well. The animals were returned to their home cages after exposure to either thirdhand ENDS aerosols or control towels. At the end of the third week, animals were properly euthanized, BALF was obtained, and lungs, brain, blood, and liver were removed for future gene expression studies.

### PM monitoring

Air monitoring for PM was conducted after ENDS aerosol generation had ceased and during mouse exposures. We used an automated self-contained, filter-integrated sensor called the UPAS V2+ (Access Sensor Technologies, CO USA). Due to the limited number of sensors, we conducted PM measurements for a total of four sessions over the three-week period. Thus, PM was measured in a mouse cage with thirdhand ENDS aerosol contaminated towel as well as in a mouse cage with control towels for a couple of hours at one-second intervals.

### Primary outcome: lung and blood protein profiling

Approximately 100 μL of serum and BALF from the mice used in this study were sent to Eve Technologies Corp (Calgary, Canada) to assess cytokine and chemokine expression. The multiplexing analysis was performed using the Luminex^™^ 200 system (Luminex, Austin, TX, USA) by Eve Technologies Corp. Eighteen markers were simultaneously measured in the samples using Eve Technologies’ Mouse High Sensitivity 18-Plex Discovery Assay^®^ (MilliporeSigma, Burlington, Massachusetts, USA) according to the manufacturer’s protocol. The 18-plex consisted of GM-CSF, IFNγ, IL-1α, IL-1β, IL-2, IL-4, IL-5, IL-6, IL-7, IL-10, IL-12(p70), IL-13, IL-17A, KC/CXCL1, LIX, MCP-1, MIP-2 and TNFα. The assay sensitivities of these markers ranged from 0.06–9.06 pg/mL for the 18-plex, and samples were run in duplicates.

### Secondary outcomes: organ weights, mouse weights and tissue biomarkers

Animals were weighed twice a week during the three-week period to ensure that they were not losing or gaining weight abnormally. At the time of tissue harvest, whole lungs, livers, and brains were quickly weighed before flash freezing. Serum, lung, and liver tissue were sent to the Clinical Pharmacology Analytical Core at Indiana University to measure levels of nicotine, cotinine, 3-OH-cotinine, and cotinine-n-oxide using an HPLC-MS/MS (Sciex 6500 + QTRAP). Samples were run in duplicates.

### Organic chemical analysis

Three types of towel samples were assessed for chemicals during each analytical run. First, 1” by 1” samples were cut from the main towel without previous exposure to ENDS or mice (blank). Then, the middle sections of all control towels and exposed towels were also cut for testing. All samples were analyzed by headspace-GC-Time-of-Flight on an Agilent 7890B/7250 GC-Quadrupole Time-of-Flight (QToF) mass spectrometer system at the Mass Spectrometry Facility at Indiana University. The “1 × 1” square cotton towels were placed in 10 mL glass headspace vials from Gerstel US with tweezers and capped with PTFE/Silicone magnetic screw caps from Restek. Sample incubation and introduction was done via a Gerstel MPS Robotic Pro system equipped with a 2.5 mL heated headspace syringe tool. Samples were transferred via the Gerstel robotic and incubated in a Gerstel agitator oven at 200 °C for 30 min with a pattern of 10 seconds of shaking followed by 1 second of stationary positioning before resuming shaking at 250 rpm. Following incubation, the robotic syringe (held at 150 °C) sampled 2.5 mL of headspace vapor and injected into a Gerstel Cooled Injection System 4 (CIS-4) PTV inlet. The CIS-4 was held at −80 °C for 0.5 min to cryofocus the analytes onto the head of the GC column and then was heated at 12 °C/second to 250 °C and then held at that temperature for 2 min to begin the temperature program. The sample was then injected onto an Agilent DB-5MS column (30 m × 250 μm × 0.25 μm) operating at 1.4 mL/min constant flow with a 5:1 split ratio. Temperature programming was as follows: The column was initially held at 35 °C for 2.5 min and then ramped at 18 °C/min to 125 °C at which point the ramp rate was changed to 30 °C/min to a final temperature of 280 °C and then held for 2.33 min for a total runtime of 15 min. The QToF quadrupole was operated in a scanning mode and was set to pass masses above m/z 30 and the mass range was defined to be from m/z 20 to 400 with a data collection rate of 6 Hz.

Initial data were processed in Masshunter Qual Browser version 10.0. Molecular features were identified by the Find by Molecular Feature algorithm. Potential molecular features were then compared against the NIST2020 database with a requirement of a matching score of at least or better than 80.0, followed by manual inspection of compounds *vs*. NIST2020 entries. Putative compounds identified in this manner were then filtered against putative compounds identified in control towels, and only putative identities that were unique to exposed towels were then selected for quantitation. Putative compounds that showed statistically significant changes were then confirmed via authentic standards. Aliquots of authentic standards were applied to clean cotton towels and then treated in the same manner as study samples to account for any effects due to the process of volatilization of compounds on the towels. Quantitation was performed using Agilent MassHunter Workstation Quantitative Analysis for ToF version 10.0.

### Data analysis

Descriptive statistics and time series plots were used to assess real-time PM data. Wilcoxon signed-rank tests were employed to assess whether the difference between control and exposed PM cage measurements had a mean signed rank of 0, as it is more robust against outliers and heavy tail distributions.

Secondly, for the GC mass spectrometry data, we only employed AUC values for compounds with > 95% putative identity based on their signals. We encountered challenges with further quantification, so to assess which chemicals were identified with more frequency on control *vs*. ENDS-contaminated towels, we calculated ratios of the AUC values for chemicals detected in each sample. As chemical analyses were run in triplicates, we used the average of the three samples after blank towel sample subtraction. We first assessed how frequently a chemical was detected in ENDS-contaminated towel samples compared to control samples. Then, the ratios of AUC values were uploaded in MetaboAnalyst 5.0^[18]^ for univariate and machine learning approaches to identify chemicals that were differentially detected on exposed *vs*. control towels.

For the protein data, we employed one-way analysis of variance (ANOVA) to compare thirdhand ENDS *vs*. control mice with the following parameters:

- If there were missing concentration values due to the fluorescence being out of range or below the limit of detection, markers were excluded.
- If significance was reached (< 0.1), a three-way ANOVA was performed to understand the impact of thirdhand ENDS exposure on mouse age and sex.

Protein values were natural log-transformed for the ANOVA. We also conducted an analysis to evaluate secondary outcomes such as cell counts, organ weights, and weekly mice weight measurements to determine the impact of thirdhand ENDS exposure. For cell counts and organ weights, we used ANOVA, and for the animal weights, we employed linear mixed-effects models similar to the equation (1) below.

(1) weight~exposure status+sex+time+exposure status*time(1|ID)

where *weight* is the mouse weight in grams measured twice a week, *exposure status* is whether the mouse was exposed to ENDS-contaminated or control towels, *sex* is whether the mouse was male or female, *time* is when the weight was taken (from 0 which was baseline until the last measurement was taken at the time of sacrifice), exposure status*time is the interaction between exposure status and time. The model includes a random intercept for mouse subject *ID*.

We summarize data as mean ± standard deviation (sd). The remainder of the analysis was performed in R^[19]^ with the following packages: *ggpubr*, *ggplot2* and *dplyr*. Statistical significance was determined by *P* values of < 0.05, while *P* values that were < 0.1 were considered to be marginally significant.

## RESULTS

### Particulate matter measurements

**Figure 2.**
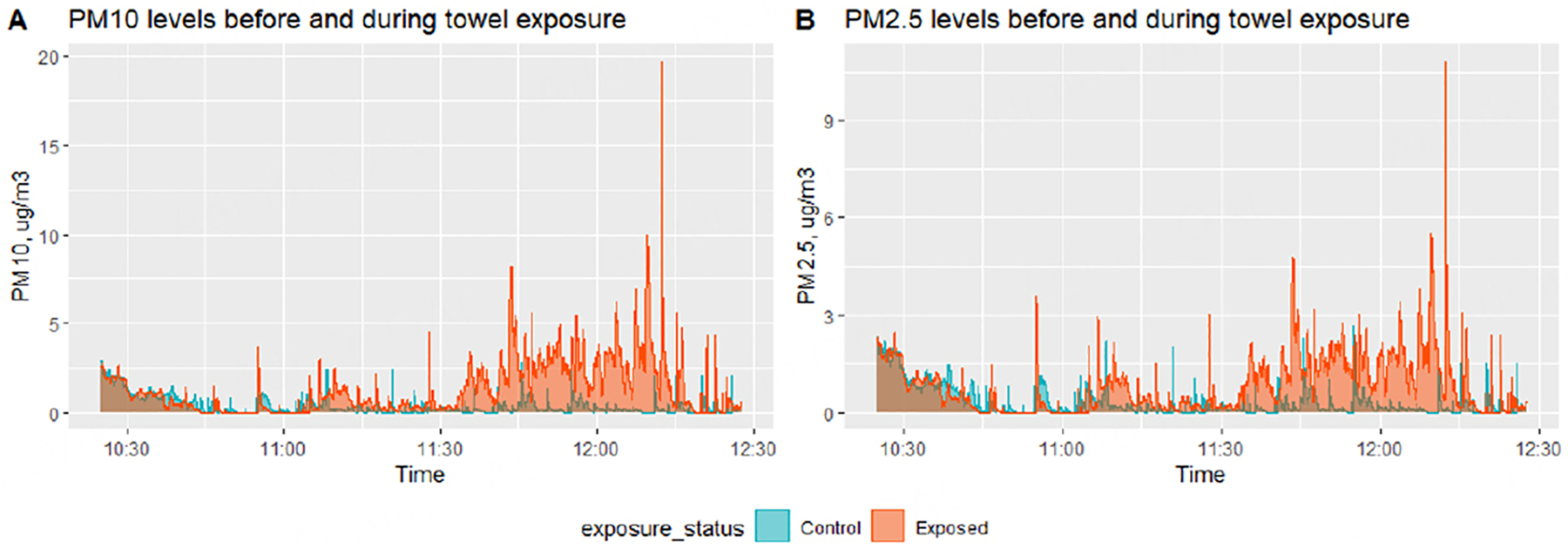
Time series plots of real-time PM_10_ (A) and PM_2.5_ (B) measurements taken during one of the experimental periods. Real-time samplers were turned on but programmed to start logging data around 9:45am. Air samplers were placed side-by-side on a lab bench with one or two personnel working in the room. Towel samples were exposed to ENDS aerosols for one hour from ~ 9:45 am till ~ 10:45 am, while controls were kept in an airtight Ziplock bag. Then, ENDS-contaminated towels were also placed in airtight Ziplock bags and transported to the vivarium by 11:15 am. New mouse cages were set up, and towels and samplers were placed in the cages before introducing the respective group of mice into the new cages by 11:25 am. Then, at approximately 12:25 pm, air samplers were turned off, mice were placed in their home cages and the towels were placed back into the Ziplock bags.

Real-time PM levels were measured during four different monitoring sessions lasting for 2–3 h. Summary statistics data presented are for each second during the one-hour time points that mice were actually introduced on the towels. The mean (sd) PM_10_ and PM_2.5_ measurements in the cage with towels were 8.3 (14.0) and 4.6 (7.5) μg/m^3^, respectively. Maximum values of 137.6 and 74.4 μg/m^3^ were recorded, respectively, during four different air quality monitoring sessions. On the other hand, in the control cages, mean (sd) PM_10_ and PM_2.5_ were 6.1 (11.2) and 3.7 (6.6) μg/m^3^. Maximum values of 76.1 and 45.1 μg/m^3^ were recorded, respectively, in control cages. In general, when the animals were active on the towels, there were spikes in PM measurements, with the cage with ENDS-contaminated consistently having higher PM levels [Supplementary Figure 1]. Figure 2 provides an example of a time series of events during one experimental setup. Please note that single PM measurement data may not be representative of actual PM levels in the cages. It starts from the time thirdhand ENDS aerosols are generated onto the towels, towels transported to the animal facility (located in the basement of the same building), when animals are exposed to the towels for an hour, until air samplers are turned off a few minutes after the last animal is removed from the experimental cage. Wilcoxon signed-rank test results revealed *P* values < 0.001 when both PM_10_ and PM_2.5_ measurements in the exposed cages were compared with control cages.

### Organic chemicals

**Figure 3.**
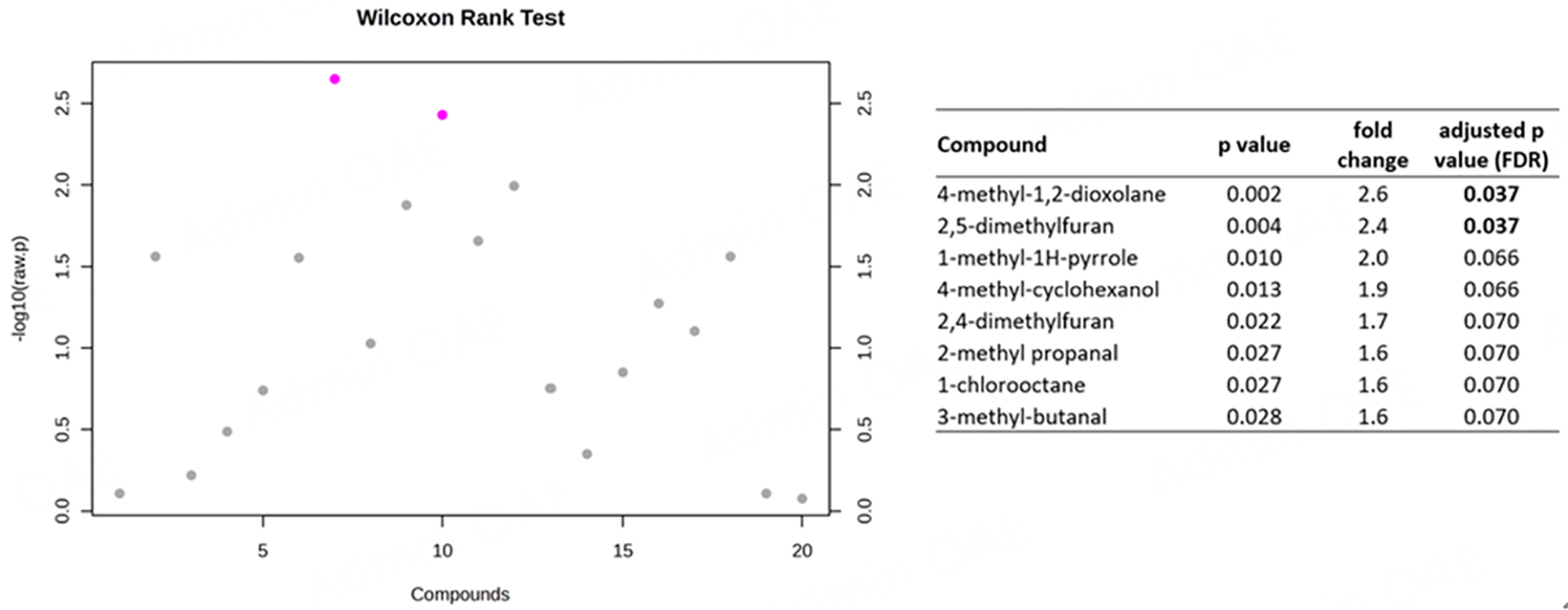
Wilcoxon rank sum tests of proportions of chemicals identified in ENDS contaminated *vs*. control towels. Test statistics are provided for chemicals with ≥ 1.5-fold change higher proportion in ENDS-contaminated towels compared to control towels. Compounds with pink dots are > 2.0-fold change with *P* values < 0.05. ENDS: electronic nicotine delivery systems.

**Figure 4.**
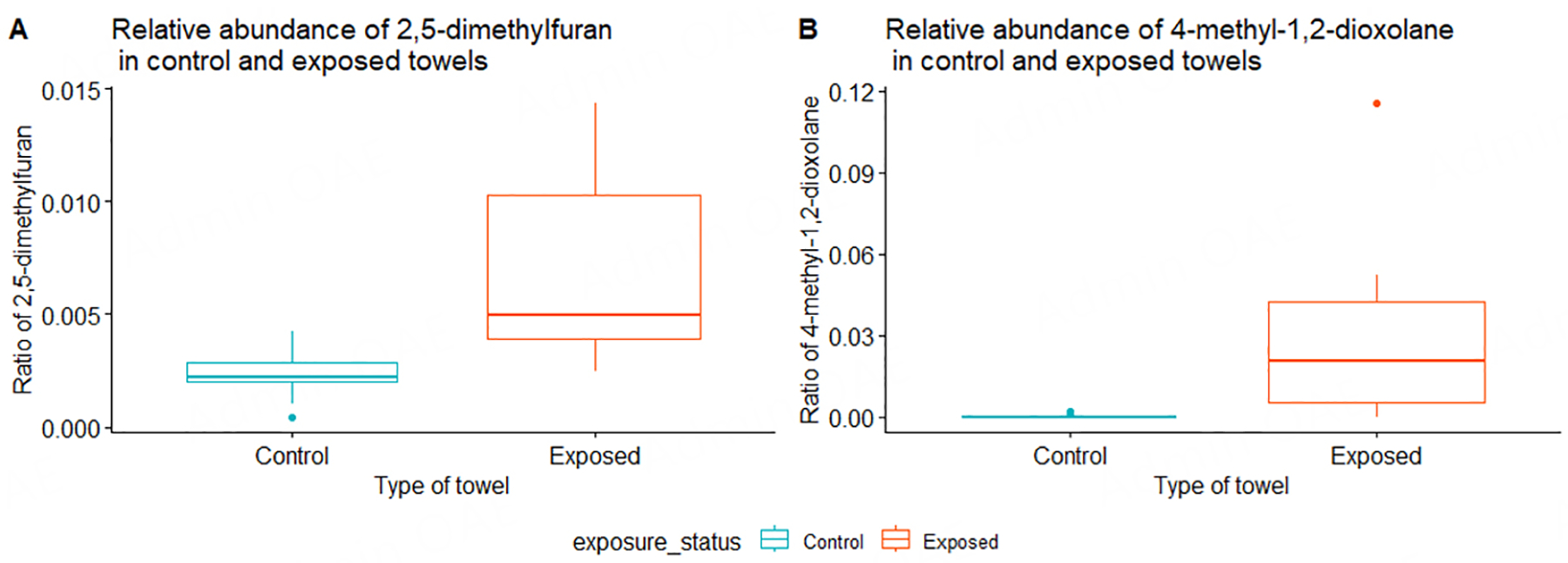
Boxplots of proportions (or ratios) of 2,5-dimethylfuran (A) and 4-methyl-1,2-dioxolane (B) identified in ENDS-contaminated *vs*. control towels. These ratios are derived from the AUC values for chemicals detected in each sample towel sample. ENDS: electronic nicotine delivery systems.

To help determine whether thirdhand ENDS exposure occurred, we looked at which organic chemicals were found on the contaminated towels in comparison to control towels and in the vape fluid from which aerosols were generated. While the vape fluid mostly contained nicotine [Table 1A], two other chemicals that were in the vape fluid were also detected in the ENDS-contaminated towels: 4-methyl-1,2-dioxolane and 4-methyl-cyclohexanol [Table 1B]. None of these two were found in the control towels [Table 1B]. Other chemicals such as 2,5-dimethylfuran, 2,4-dimethylfuran, and hexanal were found in ENDS-contaminated towel samples, but not in control towel samples [Table 1C]. Similarly, the proportion of chemicals such as 2-methyl propanal, 3-methyl-butanal, and 1-methyl-1H-pyrrole were also found in relatively higher proportion in ENDS-contaminated towel samples, in comparison to control samples [Table 1D]. Other chemicals were at the same proportion or lower in ENDS-contaminated (exposed towels) compared to controls, except for 2-aminocyanoacetamide, which is also found in all samples, including vape fluid, albeit at various proportions [Table 1E]. Wilcoxon rank tests of these chemicals between ENDS-contaminated towels and controls revealed statistically significant differences in two chemicals: 2,5-dimethylfuran and 4-methyl-1,2-dioxolane [Figure 3]. Interestingly, both compounds were detected in higher proportions in ENDS-contaminated towels *vs*. control towels [Figure 4A and 4B].

### Serum and BALF proteins

**Figure 5.**
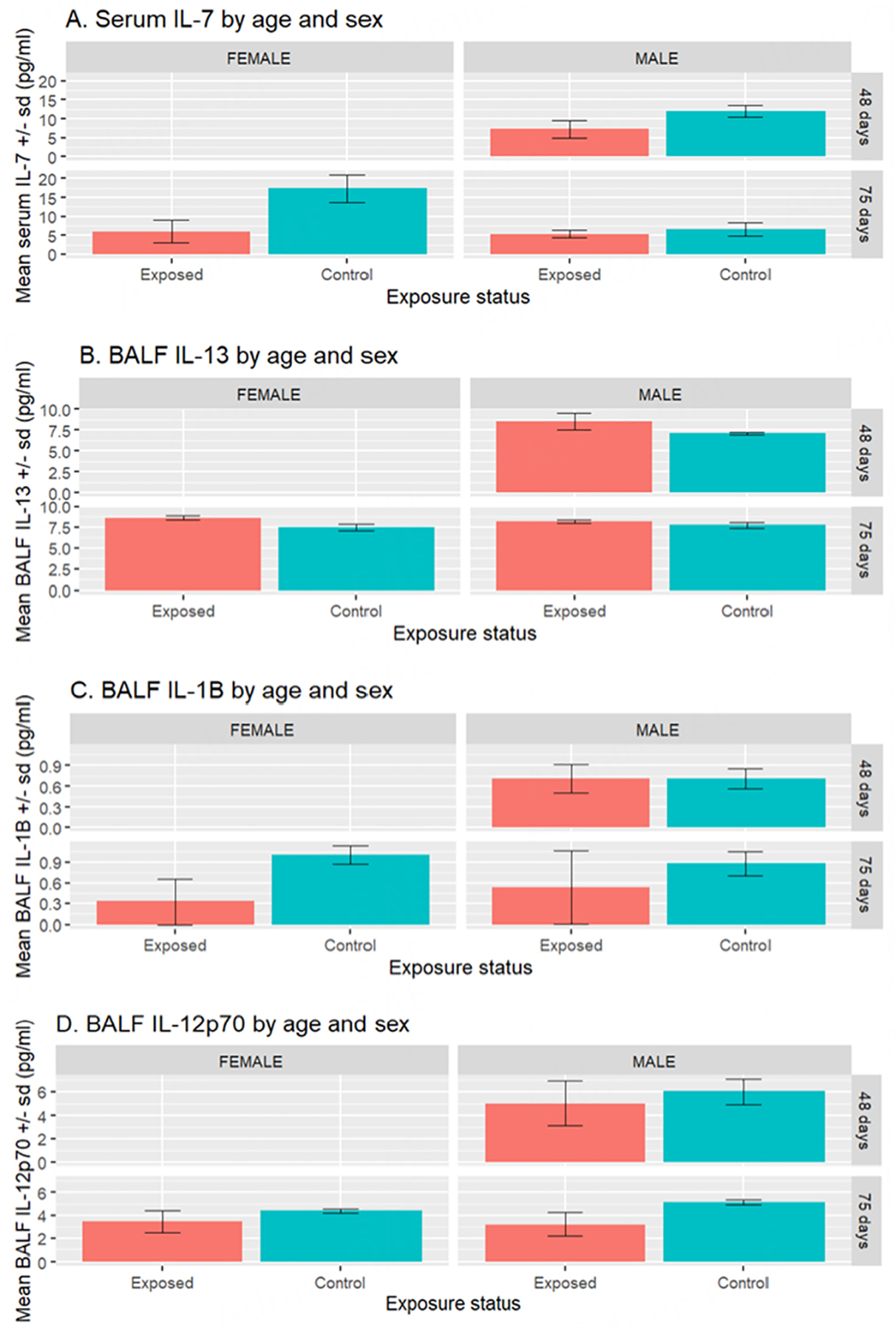
Mean (± standard deviation) of Il-7 levels in serum (A) and Il-13, Il-1β and Il-12p70 levels in (BALF, B, C, and D, respectively) of male and female mice exposed to towels with ENDS aerosol contamination. *n* = 2 each for all males and females in the exposed and control groups at 75 days. *n* = 3 and *n* = 2 for exposed and control males, respectively, at 48 days at the time of sacrifice. BALF: bronchoalveolar lavage fluid; ENDS: electronic nicotine delivery systems.

Summary statistics of the multiplex protein assays are seen in Tables 2 and 3. Protein results showed lower levels of Il-7 in the serum of mice exposed to ENDS-contaminated towels in comparison to control mice (*P* = 0.022) [Table 2 and Figure 5A]. When mouse age and sex are added to the model, exposure status remains significant (*P* = 0.010) while age is at marginal significance (*P* = 0.060) [Supplementary Table 1]. Il-13 levels were higher in the BALF of exposed mice, compared to controls (*P* = 0.006) [Table 3 and Figure 5B], even after adjusting for sex and age (*P* = 0.017). On the other hand, Il-1β and Il-12p70 levels in BALF of exposed mice had a trend towards lower expression, particularly among the older mice [75 days (11 weeks) old at sacrifice] [Figure 5C and D]; however, this did not reach statistical significance after adding mouse age and sex to the models (*P* ≥ 0.1) [Supplementary Table 1].

### Secondary outcomes

Most of the plasma samples assessing for nicotine and its metabolites were below the limit of quantification (LOQ) apart from two exposed mice [48 days (7 weeks) old]. Of those two mice, one had 6.3 ng/mL of cotinine in the plasma and the other had 2.5 ng/mL 3-OH-cotinine and 47.8 ng/mL of cotinine-n-oxide [Table 4]. Both mice were housed in the same cage with a third mouse and were all males from the same litter. Both mice were typically observed to be fighting after the first week of exposure to the ENDS-contaminated towel. Organ weights did not significantly differ between exposed and control mice [Table 4]. Different cell types in the BALF, except for macrophages which were marginally significantly different between control and exposed mice (*P* = 0.085), neutrophils, eosinophils, and lymphocytes did not significantly differ between exposed and control mice [Table 4]. The total number of cells per mL in the BALF was marginally significant between exposed and controlled mice (*P* = 0.060). Finally, linear mixed-effects models indicated that, overall, exposure to ENDS-contaminated towels was negatively associated with mouse weights (estimate = −3.62, *P* = 0.019) [Supplementary Table 2].

## DISCUSSION

In this study, we used a mouse exposure model to understand the local (lung) and systemic effects (blood) of thirdhand vaping exposures from the use of ENDS. Our results indicate that the average particulate matter measurements in a mouse cage with an ENDS-contaminated towel were significantly higher than in a cage with a control towel. Two compounds, 4-methyl-1,2-dioxolane and 4-methyl-cyclohexanol, were detected in vape fluid and on ENDS-contaminated towels, but not on control towel samples. Then 2,5-dimethylfuran, 2,4-dimethylfuran, and hexanal were found only in ENDS-contaminated towel samples, possibly due to novel combustion byproducts or chemical transformations. It is possible that additives (e.g., bleach or other chemicals used during towel manufacturing) on the towels may have reacted with some of the ENDS compounds to generate the above-listed chemicals. Future studies are needed to understand the mechanisms by which such chemicals were formed.

Incidentally, one of the compounds identified in our study, 2,5-dimethylfuran, has been proposed as a potential biomarker for smoking since its concentrations in blood accurately identified the smoking status of study subjects^[20,21]^. There were also lower levels of Il-7 in serum (*P* = 0.022, *n* = 7), and higher levels of Il-13 in the BALF (*P* = 0.006, *n* = 7) of mice exposed to towels contaminated with ENDS aerosols compared to mice exposed to towels with no ENDS contamination. After adjusting for sex and age, Il-7 and Il-13 levels were still significantly associated with thirdhand vaping exposure (*P* = 0.010 and *P* = 0.017, respectively). Together, these data provide further evidence that ENDS aerosols can adhere to and contaminate surfaces, act as a potential source of thirdhand vape exposure and subsequently influence lung and systemic health.

In this experimental study, we observed that PM concentrations can be relatively higher even after active vaping has ceased compared to surfaces not exposed to ENDS aerosols. Secondly, when the surface upon which ENDS aerosols have been deposited was disturbed (due to mouse movement), more ENDS-associated chemicals may have become airborne. However, this did not mean that PM levels were increased. In addition, the PM concentrations found in our study were on the lower end of what has been reported in vape shops. For instance, Li *et al*. reported median PM2.5 (IQR) concentrations in the range of 15 (6, 145)-134 (33, 541) μg/m3, depending on the type of shop (e.g., storefront or plaza) and ventilation (e.g., natural ventilation, window A/C or rooftop A/C)^[22]^. Another study of six vape shops measured PM2.5 within a range of 15.5 to 37,500 μg/m3 during active vaping^[23]^. The authors observed that exhaled ENDS particles persisted in the air, and it is important for further studies to clarify how such particles travel and mix with the air and surfaces in the indoor environment and ultimately impact human exposure.

Additionally, there appear to be organic chemicals from the vape fluid used to generate aerosols that adhered to the towels. There were also chemicals that were only found on ENDS-contaminated towel samples but not in control towel samples or vape fluid (6 mg nicotine in 50/50 PV/VG). A possible explanation of this finding could be due to the formation of novel compounds from combustion or reaction with other compounds in the vape aerosols. Pollutants in THS from cigarette smoke can adhere to indoor surfaces, become resuspended into indoor air, or react with atmospheric species, creating novel pollutants not present in the original smoke^[24]^. Thus, chemical reactions take place from the moment tobacco smoke is produced and may last for a long time^[25]^. A recent study identified novel compounds in ENDS aerosols, which had high polarity and low vapor pressure, were solid at room temperature and easily adsorbed to indoor surfaces^[26]^. The authors attributed the presence of new nicotine adducts with several compounds including propylene glycol, and tributylamine, to the formation of radicals during puff sections at high temperatures, high power output and high volume settings^[26]^. Additionally, it has been suggested these adducts can be formed at all power outputs at which the e-cigs are operated (i.e., there is no safe threshold)^[26]^. Such physicochemical properties render them a thirdhand exposure hazard^[11]^, compared to secondhand exposure which rapidly evaporates due to the ultrafine ENDS aerosol particles^[27]^.

Our study adds to the scarce literature on thirdhand ENDS exposures and their potential to contribute to morbidity. To date, there have been only two experimental studies on a BALB/c mouse model on thirdhand ENDS exposure effects^[15,16]^. In the first study (exposure duration of 8 days), the authors found decreased serum CCL1, CCL2, CCL4, CCL7 and TNF, while CCL11 (eotaxin) levels were increased compared to controls. Surprisingly, these effects were seen in mice exposed to thirdhand ENDS without nicotine^[15]^. Then, in the subchronic study (exposure duration of 4 weeks), the authors found that thirdhand ENDS exposure had no effects on body or organ weight and limited effects on airway inflammation. In general, the current study results agree with these previous studies, particularly with regard to no differences in organ weights and potential for airway inflammation. However, the current study observed a general trend of decreased body weights after three weeks of thirdhand ENDS exposures. Although this was a small sample size, there were significant differences in mouse weights at baseline, particularly among female mice who weighed less on average [Table 4]. Further studies with larger sample sizes are needed to assess whether thirdhand ENDS exposure induces differential sex effects. Differences in vape fluid, mouse strain and length of exposure (8 days *vs*. 4 weeks *vs*. 3 weeks) may have contributed to the differences between these two studies and the current study. It has been established that there is usually a decrease in IL-7 serum levels in individuals who smoke compared with individuals who do not smoke^[28–30]^. An *in vitro* study showed for the first time that cigarette smoke affects the IL-13-induced gene signature for Th2-high asthma^[31]^. By evaluating continuous exposures to thirdhand ENDS exposures, our study sets the stage to study potential harm to children, youth, people with asthma, and anyone who is in an environment where vaping has occurred.

In terms of the implications for human exposure, the behavior of ENDS users is an important factor to consider. A novel study that traced the movement of flavor chemicals/nicotine from refill fluids to exhaled aerosol revealed an important distinction among different types of ENDS users^[32]^. One such group inhales aerosol into their lungs, thereby efficiently absorbing most of the chemicals (lung inhalers), while the other group keeps much of the aerosol in their mouths and exhales only partially depleted chemicals (mouth inhalers). Mouth inhalers, therefore, have a high potential to increase thirdhand ENDS exposures. Together, this body of work has led to the premise that the constituents emitted through ENDS use are likely to age and interact with other pollutants in a similar fashion as those in traditional cigarette aerosols and can be detrimental to health. In our study, we also assessed the impact of thirdhand ENDS exposures on mice body weight and there appears to be a negative impact on weight gain. However, longer weight monitoring and larger sample sizes may be needed to support this finding.

Our study is not without limitations. First, with regard to the confirmation and quantification of compounds, we aimed to measure the instrument’s response to seven compounds in an attempt to achieve some quantitation. To do this, solutions of all 7 standards were prepared in dichloromethane (DCM) and then diluted that solution further to achieve a concentration of approximately 10 μM of each compound. Then 1, 5 or 10 μL volume of the respective solution was applied to 3 towel pieces and dried for 30 min. We found inconsistent instrument response to the amount of standard applied to the towels. An attempt to directly measure the mixture of compounds by putting the same amount of standard containing DCM solution into the headspace vials without applying it to the towels resulted in the signals being overshadowed by the DCM such that identification was more challenging. Applying the solution to the towels did confirm the identities of the compounds. Additionally, since we only utilized AUC values for compounds with > 95% putative identity based on their signals, we may have inadvertently missed compounds that could have impacted the pulmonary and systemic health of these mice. In the future, we will pursue and refine different quantification methods for optimal results. Moreover, we will assess both organic and inorganic compounds and enhance our methods to achieve not just identification but also quantification of these chemicals.

Secondly, we were limited by a small sample size, which affected the statistical significance of our data. However, this is the first time real-time PM and protein expression in the blood and lung fluid have been studied in a thirdhand ENDS model, and our results are hypothesis-generating. The role of thirdhand ENDS aerosols in altering inflammatory markers is understudied and we present novel results using this mouse model. We expect to include larger sample sizes in future studies for each sex and continue to test our hypothesis in animals of different ages. Future studies will also focus on assessing the function of individual chemicals in the aerosols, which may cause the mice to become more active and thereby increase the PM levels in the cages. We studied systemic and airway inflammation using a mouse model with the C57BL/6 background, a strain that is less sensitive to environmental exposures than other strains such as the BALB/c. However, our study results indicated that some airway and systemic inflammation can still occur. Future studies can also focus on confirming the target gene expression of the identified proteins through transcriptomic and epigenetic approaches. Given that the only two studies on thirdhand ENDS had been in BALB/c mice, we wanted to investigate whether thirdhand ENDS effects could be observed in the C57BL/6J background. Hence, we selected one hour per day for five days to assess the effects on lung inflammation. Future studies will focus on 8-h per day exposures with different exposure durations to account for better relevance to human exposures.

Additionally, it is possible that some chemicals evaporated from the ENDS-contaminated towels and contaminated control towels since mouse cages were in the same room. However, the covers of the mouse cages were lined with filters, and we expect little to no contamination. Future studies will ensure that the experiments are carried out in identical but separate rooms. Lastly, towels were changed after each week of exposure, rather than maintaining continuous exposure (e.g., to mimic a child crawling on a carpet without regular cleaning). One hour per day was a feasible amount of time for exposure in this setting. Future studies will include longer exposure times on surfaces to better depict human vaping behavior. However, these steps are beyond the scope of this current project, and our study will pave the way for future work in improving the understanding of thirdhand ENDS exposures to inform effective intervention and prevention strategies.

In sum, our study contributes to a further understanding of the impact of ENDS use on nonusers by assessing markers of airway and systemic inflammation. Herein, we delineate how thirdhand ENDS aerosols that adhere to and contaminate surfaces can influence lung and systemic health. It is essential to elucidate the impact of ENDS aerosols, including thirdhand exposures, on lung (localized) and overall (systemic) health. These data can inform tobacco use policies and help design intervention and prevention strategies for at-risk populations.

## Supplementary Material

Thirdhand vaping exposure supplementary material

## Figures and Tables

**Table 1.** Average proportion of organic chemicals detected in vape fluid and on white cotton towels exposed to ENDS aerosols compared to white cotton towels with no ENDS aerosols

Cumulative frequency	Chemical	Control towel samples (not exposed to mice)	Control towel samples (exposed to mice)	ENDS-contaminated towel samples (not exposed to mice)	ENDS-contaminated towel samples (exposed to mice)	Vape fluid
A. Chemicals found in vape fluid only						
1	nicotine	0%	0%	0%	0%	84%
B. Chemicals found in vape fluid and exposed towels only						
2	4-methyl-1,2-dioxolane	0%	0%	1%	3%	12%
3	4-methyl-cyclohexanol	0%	0%	0%	1%	1%
C. Chemicals found only in exposed towels compared to control towels						
4	2,4-dimethylfuran	0%	0%	0%	1%	0%
5	2,5-dimethylfuran	0%	0%	0%	1%	0%
6	hexanal	0%	0%	0%	1%	0%
D. Chemicals found in higher proportion in exposed towels compared to control towels						
7	1-methyl-1H-pyrrole	2%	2%	2%	4%	0%
8	2-methyl propanal	7%	7%	2%	11%	0%
9	3-methyl-butanal	2%	2%	1%	4%	0%
E. Other detected chemicals						
10	1-chlorooctane	1%	0%	0%	1%	0%
11	1-octene	0%	0%	0%	0%	0%
12	2,3-dihydrofuran	6%	3%	2%	6%	0%
13	2,3-pentadione	3%	2%	1%	2%	0%
14	2,5,6-trimethyldecane	5%	3%	2%	3%	0%
15	2,4-Dihydroxy-2,5-dimethyl-3(2H)-furan-3-one	3%	1%	1%	2%	0%
16	2-ethyl-4-methyl-1,3-dioxolane	0%	0%	0%	0%	0%
17	2-methyl-2-pentene	1%	1%	0%	1%	0%
18	3,4-pentadienal	3%	2%	1%	2%	0%
19	5-ethyl decane	5%	3%	2%	3%	0%
20	dimethyldisulfide	0%	1%	0%	1%	0%
21	benzyl chloride	0%	0%	0%	0%	0%
22	Diazene	3%	5%	0%	5%	0%
23	furfural	44%	33%	8%	23%	0%
24	methacrolein	0%	0%	0%	0%	0%
25	methylene chloride	0%	0%	0%	0%	0%
26	2-aminocyanoacetamide	12%	31%	74%	21%	3%

Nicotine was the only chemical detected in the fluid that was also not detected on either towel sample type (1A); 1B shows chemicals that were detected in vape fluid and exposed towels; 1C consists of chemicals found only on exposed towels and 1D contains chemicals that were, on average, detected more on exposed towels compared to controls; 1E lists chemicals that were detected at equal or lower proportions compared to control towels, except for 2-aminocyanoacetamide, which was in all samples at different proportions. ENDS: electronic nicotine delivery systems.

**Table 2.** Concentrations (in pg/mL) of different proteins in serum of mice exposed to towels with e-cig aerosols compared to control mice exposed to towels with no e-cig aerosols (ENDS-contaminated *vs*. control)

Serum proteins											
Protein	Exposed					Control					*P* value (all exposed *vs*. all control)
	all exposed mice (*n* = 7)	Males (*n* = 5)	Females (*n* = 2)	48 days (*n* = 3)	75 days (*n* = 4)	all control mice (*n* = 6)	Males (*n* = 4)	Females (*n* = 2)	48 days (*n* = 2)	75 days (*n* = 4)	
GM-CSF	15.3 ± 9.6	17.8 ± 10.1	8.9 ± 5.2	13.7 ± 4.2	16.5 ± 12.9	14.3 ± 6.0	16.7 ± 5.5	9.4 ± 4.5	21.3 ± 0.2	10.8 ± 3.4	0.990
IFNγ	3.9 ± 5.3	4.9 ± 6.2	1.4 ± 0.1	2.0 ± 0.5	5.3 ± 7.1	3.2 ± 1.5	3.6 ± 1.8	2.4 ± 0.3	4.7 ± 2.3	2.5 ± 0.2	0.667
IL-1α	286.3 ± 71.2	264.4 ± 63.5	340.9 ± 76.9	287.4 ± 77.8	285.4 ± 78.0	322.1 ± 53.4	303.1 ± 52.8	360.2 ± 39.2	282.1 ± 31.6	342.1 ± 53.0	0.284
IL-1β	11.8 ± 1.8	11.3 ± 1.9	12.9 ± 0.9	12.6 ± 0.9	11.1 ± 2.1	14.3 ± 6.1	16.1 ± 6.9	10.5 ± 0.6	20.1 ± 8.7	11.3 ± 1.4	0.350
IL-10	12.4 ± 6.2	14.3 ± 6.3	7.7 ± 3.6	10.9 ± 1.2	13.5 ± 8.5	25.1 ± 30.2	31.6 ± 36.8	12.0 ± 0.2	51.4 ± 50.0	11.9 ± 1.4	0.249
IL-12p70	21.1 ± 5.2	21.3 ± 5.8	20.4 ± 5.2	17.3 ± 2.3	23.9 ± 5.1	206.5 ± 297.4	297.8 ± 337.8	23.9 ± 3.5	579.1 ± 160.8	20.2 ± 4.8	0.130
IL-13	63.2 ± 35.9	72.1 ± 37.8	41.0 ± 25.1	59.5 ± 6.5	66.0 ± 50.3	88.4 ± 34.7	89.0 ± 36.7	87.1 ± 44.7	117.6 ± 18.3	73.8 ± 32.4	0.183
IL-17A	2.5 ± 0.8	2.6 ± 1.0	2.2 ± 0.01	2.8 ± 1.2	2.2 ± 0.3	4.1 ± 3.8	5.0 ± 4.5	2.3 ± 0.4	7.3 ± 6.4	2.5 ± 0.7	0.290
IL-2	19.1 ± 29.6	19.1 ± 29.6	NA	6.6 ± 4.0	37.7 ± 48.0	14.2 ± 6.8	14.6 ± 8.6	13.5 ± 3.3	19.6 ± 3.9	11.6 ± 6.6	0.661
IL-4	0.5 ± 0.2	0.6 ± 0.2	0.3 ± 0.1	0.5 ± 0.1	0.5 ± 0.3	5.5 ± 12.4	8.0 ± 15.1	0.4 ± 0.1	15.6 ± 21.4	0.4 ± 0.1	0.332
IL-5	27.0 ± 20.8	29.5 ± 24.9	20.8 ± 3.6	15.1 ± 5.7	35.9 ± 24.5	23.4 ± 24.4	27.4 ± 30.4	15.4 ± 3.8	43.0 ± 42.3	13.6 ± 3.7	0.547
IL-6	92.0 ± 90.1	126.9 ± 82.7	4.8 ± 1.5	91.6 ± 76.3	92.3 ± 111.1	64.1 ± 119.3	94.2 ± 141.8	3.9 ± 1.3	183.0 ± 169.4	4.6 ± 1.1	0.319
IL-7	6.4 ± 2.1	6.5 ± 2.0	6.0 ± 3.0	7.3 ± 2.3	5.7 ± 1.9	12.0 ± 5.2	9.3 ± 3.4	17.4 ± 3.6	12.0 ± 1.6	12.0 ± 6.68	**0.022**
KC	332.7 ± 207.9	416.1 ± 179.3	124.4 ± 96.2	389.7 ± 229.8	290.0 ± 213.5	260.3 ± 241.9	345.4 ± 261.4	90.0 ± 28.3	519.3 ± 278.5	130.7 ± 67.8	0.512
LIX	3444.7 ± 1062.7	3781.4 ± 1090.9	2603.1 ± 181.5	3206.3 ± 999.3	3623.6 ± 1222.0	3937.5 ± 1702.8	4109.8 ± 2167.6	3592.7 ± 211.2	2431.9 ± 289.1	4690.2 ± 1593.1	0.611
MCP-1	206.9 ± 149.8	262.9 ± 140.5	66.9 ± 28.9	247.8 ± 163.8	176.2 ± 155.2	130.0 ± 120.1	170.3 ± 132.4	49.4 ± 4.2	282.1 ± 43.4	54.0 ± 16.7	0.286
MIP-2	72.5 ± 9.3	73.8 ± 11.1	69.2 ± 0.0	70.9 ± 14.5	73.7 ± 5.4	82.8 ± 11.0	87.0 ± 11.0	74.5 ± 6.1	90.9 ± 3.9	78.8 ± 11.5	0.104
TNFα	21.5 ± 7.5	22.2 ± 8.6	19.6 ± 6.1	21.9 ± 5.2	21.2 ± 9.7	29.6 ± 26.2	36.5 ± 30.6	16.0 ± 8.5	60.5 ± 21.2	14.2 ± 6.9	0.891

NA means protein measurements were below the limit of detection. Results are presented as mean ± standard deviation (sd) for all exposed (*n* = 7) and all control (*n* = 6) mice. Under each exposure category, mean ± sd are presented by age and sex where applicable. NA means not measured and/or not estimated. BALF: bronchoalveolar lavage fluid; ENDS: electronic nicotine delivery systems.

**Table 3.** Concentrations (in pg/mL) of different proteins in the BALF of mice exposed to towels with e-cig aerosols compared to control mice exposed to towels with no e-cig aerosols (ENDS-contaminated *vs*. control)

BALF Proteins											
Protein	Exposed					Control					*P* value (all exposed *vs*. all control)
	all exposed mice (*n* = 7)	Males (*n* = 5)	Females (*n* = 2)	48 days (*n* = 3)	75 days (*n* = 4)	all control mice (*n* = 6)	Males (*n* = 4)	Females (*n* = 2)	48 days (*n* = 2)	75 days (*n* = 4)	
GM-CSF	NA	NA	NA	NA	NA	NA	NA	NA	NA	NA	NA
IFNγ	0.3 ± 0.2	0.4 ± 0.2	0.2 ± 0.2	0.5 ± 0.2	0.2 ± 0.1	0.3 ± 0.1	0.3 ± 0.1	0.3 ± 0.1	0.3 ± 0.1	0.2 ± 0.1	0.893
IL-1α	59.0 ± 19.0	67.5 ± 14.7	37.9 ± 6.9	69.0 ± 17.2	51.5 ± 18.7	62.7 ± 12.6	58.6 ± 13.6	70.9 ± 6.5	68.1 ± 13.4	60.0 ± 13.3	0.582
IL-1β	0.6 ± 0.3	0.6 ± 0.3	0.3 ± 0.3	0.7 ± 0.2	0.4 ± 0.4	0.9 ± 0.2	0.8 ± 0.2	1.0 ± 0.1	0.7 ± 0.1	0.9 ± 0.1	0.090
IL-10	1.7 ± 0.5	1.8 ± 0.5	1.5 ± 0.2	2.02 ± 0.6	1.4 ± 0.2	2.0 ± 0.4	2.1 ± 0.4	1.8 ± 0.4	2.3 ± 0.1	1.8 ± 0.4	0.225
IL-12p70	4.1 ± 1.5	4.3 ± 1.7	3.5 ± 1.0	5.04 ± 1.9	3.4 ± 0.8	5.2 ± 0.9	5.6 ± 0.8	4.4 ± 0.2	6.01 ± 1.0	4.8 ± 0.5	0.096
IL-13	8.5 ± 0.6	8.4 ± 0.7	8.6 ± 0.2	8.5 ± 1.0	8.4 ± 0.3	7.5 ± 0.4	7.4 ± 0.4	7.5 ± 0.4	7.1 ± 0.2	7.6 ± 0.3	**0.006**
IL-17A	0.1 ± 0.1	0.1 ± 0.1	0.1 ± NA	0.1 ± 0.1	0.1 ± 0.1	0.1 ± 0.03	0.1 ± 0.03	0.0 ± NA	0.1 ± 0	0.1 ± 0.04	NA
IL-2	6.9 ± 2.7	8.0 ± 2.3	4.1 ± 0.93	9.1 ± 2.5	5.2 ± 1.4	7.0 ± 1.8	7.3 ± 2.2	6.6 ± 1.0	8.9 ± 1.7	6.1 ± 1.0	0.731
IL-4	0.1 ± 0.07	0.1 ± 0.03	0.1 ± 0.01	0.1 ± 0.03	0.1 ± 0.03	0.1 ± 0.03	0.1 ± 0.03	0.1 ± 0.04	0.1 ± 0.01	0.1 ± 0.03	0.166
IL-5	1.0 ± 0.2	1.0 ± 0.2	1.0 ± 0.05	1.0 ± 0.2	0.9 ± 0.2	1.0 ± 0.3	0.9 ± 0.3	1.2 ± 0.2	0.9 ± 0.02	1.1 ± 0.3	0.731
IL-6	1.9 ± 1.2	2.1 ± 1.3	1.6 ± 1.0	2.0 ± 1.8	1.9 ± 0.7	2.3 ± 1.8	2.7 ± 2.1	1.6 ± 0.7	3.3 ± 3.2	1.8 ± 0.9	0.718
IL-7	0.9 ± 0.4	0.9 ± 0.5	1.0 ± 0.3	1.1 ± 0.5	0.8 ± 0.3	1.3 ± 0.7	1.3 ± 0.7	1.3 ± 0.9	1.4 ± 1.2	1.3 ± 0.5	0.272
KC	4.1 ± 0.8	4.2 ± 0.9	3.8 ± 0.5	3.9 ± 0.8	4.23 ± 0.8	4.4 ± 1.5	4.9 ± 1.7	3.5 ± 0.1	4.32 ± 2.4	4.51 ± 1.3	0.766
LIX	9.2 ± 5.8	9.2 ± 5.8	NA	11.4 ± 4.5	2.4 ± NA	10.5 ± 4.8	9.2 ± 4.7	13.0 ± 5.2	5.37 ± 1.4	13.0 ± 3.3	0.582
MCP-1	11.2 ± 4.6	11.7 ± 5.0	10.1 ± 4.7	15.0 ± 1.4	8.5 ± 4.0	14.5 ± 4.5	15.9 ± 5.1	11.7 ± 0.5	19.4 ± 3.6	12.1 ± 2.3	0.218
MIP-2	84.3 ± 12.6	87.8 ± 10.8	75.7 ± 16.7	85.8 ± 14.0	83.2 ± 13.5	96.3 ± 15.3	90.6 ± 16.0	107.7 ± 4.2	87.2 ± 18.9	100.8 ± 13.7	0.175
TNFα	1.7 ± 0.3	1.7 ± 0.4	1.5 ± 0.2	1.9 ± 0.3	1.5 ± 0.2	1.7 ± 0.4	1.6 ± 0.4	1.9 ± 0.1	1.7 ± 0.6	1.7 ± 0.3	0.943

NA means protein measurements were below the limit of detection. Results are presented as mean ± standard deviation (sd) for all exposed (*n* = 7) and all control (*n* = 6) mice. Under each exposure category, mean ± sd are presented by age and sex where applicable. *P* values are the results of one-way ANOVA, where bolded and underlined *P* values are ≤ 0.05 and < 0.1, respectively. NA means not measured and/or not estimated. BALF: bronchoalveolar lavage fluid; ENDS: electronic nicotine delivery systems; ANOVA: analysis of variance.

**Table 4.** Body, organ and BALF cell data of mice exposed to towels with e-cig aerosols compared to control mice exposed to towels with no e-cig aerosols (ENDS-contaminated *vs*. control)

	Exposed					Control					
Characteristic	all exposed mice (*n* = 7)	Males (*n* = 5)	Females (*n* = 2)	48 days (*n* = 3)	75 days (*n* = 4)	All control mice (*n* = 6)	Males (*n* = 4)	Females (*n* = 2)	48 days (*n* = 2)	75 days (*n* = 4)	*P* value
Start weight (g)	19.1 ± 2.0	20.2 ± 1.1	16.5 ± 0.71	21.0 ± 0.0	17.8 ± 1.5	22.7 ± 2.3	22.5 ± 3.0	23.0 ± 0.0	20.0 ± 1.4	24.0 ± 1.2	**0.017**
End weight (g)	21.4 ± 2.6	22.6 ± 2.07	18.5 ± 0.7	24.0 ± 1.0	19.5 ± 1.3	24.3 ± 1.8	24.8 ± 2.1	23.5 ± 0.7	23.5 ± 2.1	24.8 ± 1.7	**0.043**
Liver (g)	1.1 ± 0.1	1.2 ± 0.08	1.0 ± 0.01	1.2 ± 0.1	1.0 ± 0.1	1.1 ± 0.2	1.2 ± 0.2	0.8 ± 0.1	1.3 ± 0.1	1.0 ± 0.2	0.604
Lung (g)	0.4 ± 0.1	0.4 ± 0.06	0.3 ± 0.01	0.4 ± 0.1	0.4 ± 0.04	0.4 ± 0.1	0.4 ± 0.1	0.4 ± 0.1	0.3 ± 0.1	0.4 ± 0.1	0.851
Brain (g)	0.4 ± 0.04	0.4 ± 0.04	0.5 ± 0.001	0.4 ± 0.03	0.5 ± 0.03	0.5 ± 0.02	0.5 ± 0.03	0.4 ± 0.02	0.4 ± 0.03	0.5 ± 0.02	0.306
Nicotine (ng/mL)	NA	NA	NA	NA	NA	NA	NA	NA	NA	NA	NA
Cotinine (ng/mL)	6.3 ± NA	6.3 ± NA	NA	6.3 ± NA	NA	NA	NA	NA	NA	NA	NA
3-OH Cotinine (ng/mL)	2.5 ± NA	2.5 ± NA	NA	2.5 ± NA	NA	NA	NA	NA	NA	NA	NA
Cotinine-N-Oxide (ng/mL)	47.8 ± NA	47.8 ± NA	NA	47.8 ± NA	NA	NA	NA	NA	NA	NA	NA
Total number of cells per ml in the BALF	1.7E + 05 ± 1.1E + 05	1.9E + 05 ± 1.1E + 05	1.2E+05 ± 1.6E + 05	1.9E+05 ± 1.0E + 05	1.5E + 05 ± 1.4E + 05	5.8E + 05 ± 6.7E + 05	6.8E + 05 ± 8.2E + 05	3.6E + 05 ± 3.0E + 05	1.1E + 06 ± 1.2E + 06	3.3E + 05 ± 1.8E + 05	0.060
Eosinophil count	2.9E + 02 ± 7.5E + 02	4.0E + 02 ± 8.9E + 02	1.8E+01 ± 2.5E + 01	0	5.1E + 02 ± 9.9E + 02	4.4E + 02 ± 7.2E + 02	6.6E + 02 ± 8.2E + 02	0	8.4E + 02 ± 1.2E + 03	2.4E + 02 ± 4.9E + 02	0.716
Lymphocyte count	8.1E + 03 ± 7.6E + 03	9.5E + 03 ± 8.2E + 03	4.8E + 03 ± 6.5E + 03	6.1E + 03 ± 2.5E + 03	9.7E + 03 ± 1.0E + 04	2.5E + 04 ± 3.0E + 04	3.2E + 04 ± 3.5E + 04	1.2E + 04 ± 1.6E + 04	5.1E + 04 ± 4.4E + 04	1.2E + 04 ± 1.2E + 04	0.175
Macrophage count	1.5E + 05 ± 1.1E + 05	1.7E + 05 ± 1.0E + 05	1.1E + 05 ± 1.4E + 05	1.8E + 05 ± 1.0E + 05	1.4E + 05 ± 1.2E + 05	5.7E + 05 ± 5.7E + 05	6.1E + 05 ± 7.4E + 05	5.0E + 05 ± 4.9E + 04	9.6E + 05 ± 1.1E + 06	3.7E + 05 ± 1.4E + 05	0.085
Neutrophil count	6.9E + 02 ± 1.1E + 03	7.9E+02 ± 1.3E+03	4.4E+02 ± 4.8E+02	2.5E+02 ± 4.3E+02	1.0E+03 ± 1.3E+03	3.1E + 03 ± 3.6E + 03	4.7E + 03 ± 3.5E + 03	0	4.0E+03 ± 3.3E+03	2.7E + 03 ± 4.2E + 03	0.117

Results are presented as mean ± standard deviation (sd) for all exposed (*n* = 7) and all control (*n* = 6) mice. Under each exposure category, mean ± sd are presented by age and sex where applicable. Please note that only starting and ending mouse ages are reported in this table for readability. *P* values are the results of one-way analysis of variance (ANOVA) comparing control and exposed mice, where bolded and underlined *P* values are ≤ 0.05 and < 0.1, respectively. NA means not measured and/or not estimated.

## Data Availability

Data can be retrieved here: https://github.com/exposurelabiu/thirdhand-pilot-August-2023.git
