# Supplementary material for: Thirdhand vaping exposures are associated with pulmonary and systemic inflammation in a mouse model
Source: J Environ Expo Assess. Author manuscript; Available in PMC 2024 May 13. (PMC11090496; doi:10.20517/jeea.2023.27)
Supplement: Thirdhand vaping exposure supplementary material [file NIHMS1991515-supplement-Thirdhand_vaping_exposure_supplementary_material.pdf]

**Supplementary Material**

# Thirdhand vaping exposures are associated with pulmonary and systemic inflammation in a mouse model

**Sarah Commodore[1], Shikha Sharma[1], Carolyn Damilola Ekpruke[1], Robert Pepin[2], Angela M. Hansen[2], Dustin Rousselle[1], Maksat Babayev[1], Jonas M. Ndeke[3], Rachel Alford[1], Erik Parker[4], Stephanie Dickinson[4], Sunita Sharma[5], Patricia Silveyra[1,6]**

[1]Department of Environmental and Occupational Health, School of Public Health Bloomington, Indiana University, Bloomington, IN 47408, USA.

[2]Department of Chemistry, Indiana University, Bloomington, IN 47405, USA.

[3]Department of Epidemiology and Biostatistics, School of Public Health Bloomington, Indiana University, Bloomington, IN 47405, USA.

[4]Biostatistics Consulting Center, Department of Epidemiology and Biostatistics, School of Public Health Bloomington, Indiana University, Bloomington, IN 47405, USA.

[5]Division of Pulmonary Sciences and Critical Care Medicine, University of Colorado Anschutz Medical Campus, Aurora, CO 80045, USA.

[6]Department of Medicine, Indiana University School of Medicine, Indianapolis, IN 46202, USA.

**Correspondence to:** Dr. Sarah Commodore, Department of Environmental and Occupational Health, School of Public Health Bloomington, Indiana University, 2719 E 10th St, Bloomington, IN 47408, USA. E-mail: scommod@iu.edu

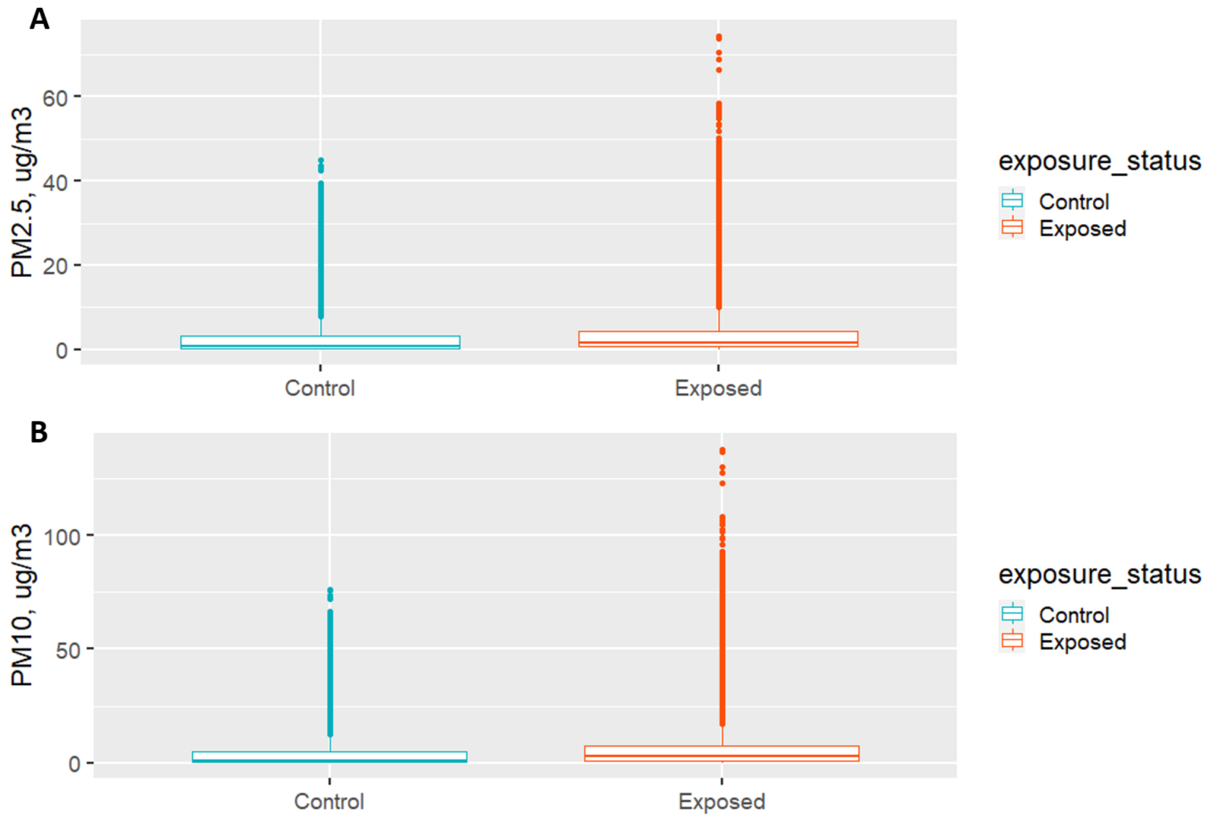


**Supplementary Figure 1.** Boxplots of combined $PM_{2.5}$ (A) and $PM_{10}$ (B) data measured during four different monitoring periods in cages with ENDS-contaminated towels and control towels.

**Supplementary Table 1. Analysis of variance (ANOVA) of Il-7 in serum and Il-13, Il-1β and Il-12p70 in bronchoalveolar lavage fluid (BALF) of mice exposed to towels with ENDS-contaminated aerosols compared to control mice**

| **Serum Il-7** | | | | | |
|---|---|---|---|---|---|
| **Parameter** | Df | Sum Sq | Mean Sq | F value | Pr(>F) |
| Exposure status | 1 | 1.12 | 1.12 | 12.31 | **0.01** |
| Sex | 1 | 0.19 | 0.19 | 2.12 | 0.19 |
| Age | 1 | 0.45 | 0.45 | 4.94 | 0.06 |
| Exposure status*Age | 1 | 0.01 | 0.01 | 0.14 | 0.72 |
| Exposure status*Sex | 1 | 0.44 | 0.44 | 4.81 | 0.06 |
| **BALF Il-13** | | | | | |
| | Df | Sum Sq | Mean Sq | F value | Pr(>F) |
| Exposure status | 1 | 0.05 | 0.05 | 9.72 | **0.02** |
| Sex | 1 | 0.00 | 0.00 | 0.24 | 0.64 |
| Age | 1 | 0.00 | 0.00 | 0.23 | 0.65 |
| Exposure status*Age | 1 | 0.00 | 0.00 | 0.94 | 0.36 |
| Exposure status*Sex | 1 | 0.00 | 0.00 | 0.72 | 0.43 |
| **BALF Il-1β** | | | | | |
| | Df | Sum Sq | Mean Sq | F value | Pr(>F) |
| Exposure status | 1 | 1.47 | 1.47 | 3.25 | 0.11 |
| Sex | 1 | 0.28 | 0.28 | 0.62 | 0.46 |
| Age | 1 | 0.13 | 0.13 | 0.29 | 0.61 |
| Exposure status*Age | 1 | 0.91 | 0.91 | 2.02 | 0.20 |
| Exposure status*Sex | 1 | 0.21 | 0.21 | 0.46 | 0.52 |
| **BALF Il-12p70** | | | | | |
| | Df | Sum Sq | Mean Sq | F value | Pr(>F) |
| Exposure status | 1 | 0.26 | 0.26 | 3.52 | 0.10 |
| Sex | 1 | 0.11 | 0.11 | 1.48 | 0.26 |
| Age | 1 | 0.19 | 0.19 | 2.58 | 0.15 |

| | | | | | |
|---|---|---|---|---|---|
| Exposure status*Age | 1 | 0.02 | 0.02 | 0.25 | 0.63 |
| Exposure status*Sex | 1 | 0.03 | 0.03 | 0.38 | 0.56 |

*P* values in bold are < 0.05, while underlined p values are < 0.1.

**Supplementary Table 2. Results of linear mixed effects model for exposed and control mouse weights over time**

| Parameter | Estimate | Standard Error | df | t value | P value |
|---|---|---|---|---|---|
| (Intercept) | 21.1 | 1.3 | 11.0 | 15.6 | **<0.0001** |
| Exposure status (exposed mice as reference) | -3.6 | 1.3 | 12.0 | -2.7 | **0.019** |
| Sex (male as reference) | 2.3 | 1.4 | 9.9 | 1.7 | 0.122 |
| Time 1 (time 0 is baseline reference of initial mouse weight) | 0.5 | 0.5 | 55.9 | 1.1 | 0.288 |
| Time 2 | 1.3 | 0.5 | 55.9 | 2.9 | **0.006** |
| Time 3 | 1.5 | 0.5 | 55.9 | 3.2 | **0.002** |
| Time 4 | 2.7 | 0.5 | 55.9 | 5.7 | **<0.0001** |
| Time 5 | 2.5 | 0.5 | 56.0 | 4.8 | **<0.0001** |
| Time 6 | 1.0 | 0.5 | 56.0 | 2.0 | 0.056 |
| Exposure status*time 1 | 0.4 | 0.6 | 55.9 | 0.6 | 0.576 |
| Exposure status*time 2 | -0.05 | 0.6 | 55.9 | -0.1 | 0.941 |
| Exposure status*time 3 | 0.2 | 0.6 | 55.9 | 0.3 | 0.737 |
| Exposure status*time 4 | -0.8 | 0.6 | 55.9 | -1.3 | 0.208 |
| Exposure status*time 5 | -0.7 | 0.7 | 56.1 | -1.0 | 0.330 |
| Exposure status*time 6 | 1.3 | 0.7 | 56.1 | 1.7 | 0.093 |
